# Review of Recent Developments of Glass Transition in PVC Nanocomposites

**DOI:** 10.3390/polym13244336

**Published:** 2021-12-10

**Authors:** Jolanta Tomaszewska, Tomasz Sterzyński, Aneta Woźniak-Braszak, Michał Banaszak

**Affiliations:** 1 Faculty of Chemical Technology and Engineering, Bydgoszcz University of Science and Technology, Seminaryjna, 85-326 Bydgoszcz, Poland; 2 Faculty of Mechanical Engineering, Poznan University of Technology, Piotrowo, 60-965 Poznan, Poland; tomasz.sterzynski@put.poznan.pl; 3 Faculty of Physics, Adam Mickiewicz University in Poznan, Uniwersytetu Poznańskiego, Wieniawski, 61-614 Poznan, Poland; abraszak@amu.edu.pl (A.W.-B.); mbanasz@amu.edu.pl (M.B.)

**Keywords:** nanocomposites, thermal analysis, thermal properties, PVC glass transition

## Abstract

This review addresses the impact of different nanoadditives on the glass transition temperature (*Tg*) of polyvinyl chloride (PVC), which is a widely used industrial polymer. The relatively high *Tg* limits its temperature-dependent applications. The objective of the review is to present the state-of-the-art knowledge on the influence of nanofillers of various origins and dimensions on the *Tg* of the PVC. The *Tg* variations induced by added nanofillers can be probed mostly by such experimental techniques as thermomechanical analysis (TMA), dynamic mechanical analysis (DMA), differential scanning calorimetry (DSC), and dielectric thermal analysis (DETA). The increase in *Tg* is commonly associated with the use of mineral and carbonaceous nanofillers. In this case, a rise in the concentration of nanoadditives leads to an increase in the *Tg* due to a restraint of the PVC macromolecular chain’s mobility. The lowering of *Tg* may be attributed to the well-known plasticizing effect, which is a consequence of the incorporation of oligomeric silsesquioxanes to the polymeric matrix. It has been well established that the variation in the *Tg* value depends also on the chemical modification of nanofillers and their incorporation into the PVC matrix. This review may be an inspiration for further investigation of nanofillers’ effect on the PVC glass transition temperature.

## 1. Introduction

Amorphous polymers have been extensively studied due to their numerous applications required by the tremendous industrial and technological growth [1,2,3]. These materials are characterized by a random disordered molecular structure [4]. Cooling the molten polymers below their equilibrium melting temperature without crystallization results in a disordered molecular structure that takes the form of a solid-like noncrystalline glass [5,6]. The reversible transformation of amorphous structures from the molten or rubber-like state into the stiff and relatively brittle glassy state, observed on cooling, is denoted as the glass transition and takes place over a temperature range characterized by the glass transition temperature (*Tg*) [7,8]. Glass transition is one of the most important physicochemical features of amorphous polymers. Below the glass transition temperature, the molecular dynamics in the polymer are restricted, which means that the molecular motion is limited to local rearrangements, such as the vibrations and rotations of atoms, in a state characteristic of solid-like materials. Above the glass transition temperature, translational movements dominate, as in the liquid-like state. The *Tg* temperature indicates a limit of the temperature-dependent applications of polymers, polymer blends, and polymer composites as its achievement is accompanied by gradual changes in various physicochemical properties, such as electrical and heat conductivity, dielectric constant, specific volume, thermal capacity, and others [9,10]. Therefore, a comprehensive analysis of the glass transition at the molecular level is crucial for determining the properties associated with the processing of new polymeric materials and with their wide applications.

Polymer composites are widely used in industrial applications due to their ability to combine the desired properties of various polymeric species. In particular, polyvinyl chloride (PVC) belongs to the most frequently used materials in the creation of nanocomposites because of its beneficial properties, such as its comparatively low cost, extensively developed processing, the possibilities of modification of its mechanical properties, and its high environmental resistance [11,12,13,14,15]. The production and application of this polymer are constantly growing worldwide. The restrictions of PVC applications are mostly related to the relatively high glass transition temperature of this polymer, resulting from strong polar interactions in the PVC molecules as a consequence of the presence of chlorine atoms [16].

It is well known that the glass transition temperature depends on the molecular structure and molecular weight of the polymer. Moreover, its thermal history depends on the measurement method and the rate of heating or cooling, as well as on many other factors, including the use of various processing aids, such as plasticizers and nanofillers [1,2,3,4,5,6,7,8]. However, the most frequently used modifiers of PVC glass transition temperature are specific plasticizers, in the form of small molecules, which are introduced into a polymer macromolecular structure and usually lead to a reduction in the *Tg* value [2,11,17,18].

Despite a large number of sources [19,20,21,22,23,24,25,26,27,28,29,30,31,32,33,34,35,36] that report on the impact of nanoadditives on the properties of PVC nanocomposites, only some of them concern the influence of nanofillers on the thermal properties of this polymer in its glassy state.

The aim of this study is to investigate and analyze the influence of various nanofillers on the processing and physical properties related to polymer vitrification, which is of interest to both industry and science.

## 2. The Behavior of Polymer Macromolecules during Glass Transition

**Figure 1:**
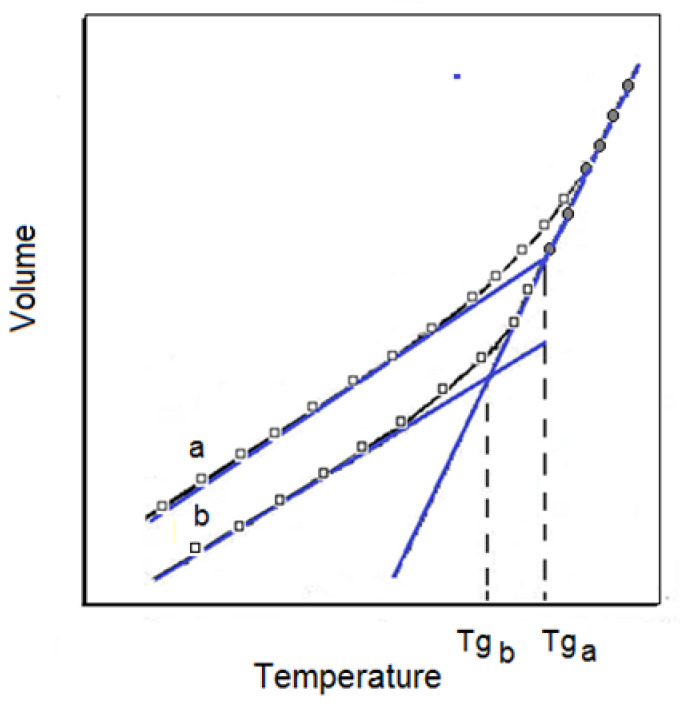
The temperature dependence of the volume of an amorphous polymer at a constant pressure. The curve **a** corresponds to a short time and **b** to a longer time after solidification [8].

As mentioned above, the reversible transformation of an amorphous polymer from the molten or rubber-like state (highly elastic) into the stiff and relatively brittle glassy state is referred to as the glass transition. This transformation, observed upon the cooling of the polymer, takes place in a strictly defined temperature range, but, for the sake of convenience, a single glass transition temperature, *Tg*, is reported. The temperature dependencies of specific volumes of two polymer samples differing in the time of solidification are presented in Figure 1. 

According to Kalogeras and Hagg Lobland [8], the upper curve exemplifies the case when this time is very short; the lower V = f (T) run represents the sample with a much longer time after solidification; thus, the influence of thermal shrinkage is obviously superior compared with the upper run. A longer cooling time evidently results in a lower specific volume and thus in a higher density, as observed for all the temperatures. For the tangents drawn for both V = f (T) curves, the points of intersection of the linear functions allowed for the identification of transition temperatures. As the cooling rate is higher, the shrinkage effect is smaller and *Tg* is higher than that the cooling rate is slower. As follows from Figure 1, a certain temperature range of the glass transition and not a single temperature point is always observed, depending in this case on the thermal history of the samples.

It has to be stressed that the glass transition temperature belongs to the most important factors that limit the temperature-dependent applications of polymer materials. Many concepts describing the behavior of macromolecules during the glass transition have been developed, including the free volume theory, the kinetic interpretation of glass transition, the thermodynamic model, and the energy landscape approach [1,2,3,4,5,6,7,8,9,10,37,38,39,40,41,42,43,44]. These theories represent three different perspectives on the same phenomenon.

The free volume theory assumes the existence of a free volume in the form of segment-size voids, which can lead to a variety of correlated and cooperative molecular motions. This theory specifies the relationship between the coefficients of expansion below and above the glass temperature transition *Tg* and also yields the Williams-Landel-Ferry (WLF) equation relating viscosity to temperature. The WLF equation is as follows [37]:

(1) $$\eta =A\;\exp\;\left[B/\left(T-T_{0}\right)\right]$$

where η is viscosity, *A* and *B* are temperature-independent constants, and *T*_0_ is the temperature at which the viscosity diverges, indicating the glass transition. A similar equation holds for the temperature dependence of relaxation times.

According to the free volume model, below the glass transition, the free volume in polymers (the part of the volume that is not occupied by macromolecules or their segments) is too small for translational motions. It is assumed that in the glassy state the polymeric system may not reach thermodynamic stability, so it exists in a metastable state, which is in agreement with the kinetic interpretation of the glass transition. The transition into the glassy state is governed by kinetic processes that can be described by the temperature-dependent relaxation time.

The kinetic theory defines *Tg* as the temperature at which the relaxation time of the segmental motions in the polymer chain is of the same order of magnitude as the time scale of the experiment. It considers the molecular and macroscopic response within a varying time frame. The temperature of the transition can be shifted by changing the time scale of the experiment, and, therefore, the measured relaxation time near the transition can be of the same order as the time scale of the experiment. Moreover, the kinetic theory provides quantitative information about the heat capacity below and above the glass transition temperature [5].

The thermodynamic theory presented by Gibbs and DiMarzio [38] conjectures the existence of a true second-order transition temperature at infinitely long time scales when the material finally reaches equilibrium. This theory introduced the concept of “a cooperatively rearranging region”. According to this idea, there is a true equilibrium second-order transition at the temperature denoted *T*_2_, which is usually significantly (from 30 K to 50 K) lower than the observed value of *Tg*. Using the method developed by Gibbs and DiMarzio, it is possible to predict or interpret the *Tg* dependences on the polymer concentration, the copolymer composition, and the degree of the polymer crosslinking. Another interesting thermodynamic approach is given by the following equation derived by Adam and Gibbs [43]:

(2) $$\tau =A\;\exp\;\left(B/\left(T\cdot Sc\right)\right)$$

where τ is the relaxation time, *T* temperature, and *Sc* is the configurational entropy related to the number of equivalent minima in the polymer’s multidimensional surface formed by the energy.

Finally, it is of practical interest that, according to Kalogeras [44], the glass transition region is usually observed in a restricted range of temperatures in which the molecular relaxation time, characteristic of the investigated system, changes by about 2 to 2.5 orders of magnitude. This characteristic time may reach a value of about 100 s at the so-called laboratory time scale, corresponding to long-range segmental motions.

The energy landscape approach is based on studying the total energy of the system as a function of all the relevant coordinates, mostly by employing advanced molecular simulations. This approach is conceptually related to the theory developed by Adam and Gibbs in which the number of energy minima is calculated [37]. This theory has an interesting extension that takes into account fluctuations in the number of molecules inside the cooperative region [45].

The development of these theories indicates both the relevance of the nature of glass transition and the insufficient (but gradually increasing) understanding of the phenomena associated with it [46]. Theoretical explanation of glass transition is still a major challenge and a central topic in contemporary physics. As Anderson [47] has stated: “the glass transition remains the deepest and most important problem in the solid-state physics”.

As regards the molecular simulation for predicting the glass transition temperature of PVC and its nanocomposites, a recent paper by Li et al. [48] and the references therein provide a comprehensive review of the most recent advances in this field. Their paper presents a variety of structural and dynamic simulation methods used to probe the glass transition and its deep nature.

## 3. *Tg* Measurement Methods

The transition of the polymer by cooling into a glassy state can be observed experimentally by calorimetric, dielectric, mechanical, and spectroscopic techniques [1,2,3,4,5,6,7,8,9,10]. The glass transition is detected by significant changes in physical, mechanical, electrical, or thermodynamic polymer properties, such as heat conductivity, dielectric constant, specific volume, refractive index, thermal capacity, and others. The glass transition is consi-dered to be a kinetic phenomenon that does not occur at a fixed temperature but covers a wide range of temperatures. As indicated in a previous section, *Tg* is used to describe the glass transition [1] as a very important property of glass-forming materials determining the industrial application and processing of these materials. There are various techniques to determine the value of *Tg*, such as thermodilatometry (TD), thermomechanical analysis (TMA), dynamic mechanical analysis (DMA), differential scanning calorimetry (DSC), temperature modulated DSC (TMDSC), dielectric relaxation spectroscopy (DRS), thermally stimulated depolarization currents (TSDC), viscosity measurements, electrical conductivity measurements, and optical methods [49,50,51,52,53,54,55,56,57,58,59,60,61,62,63,64,65,66]. It should be emphasized that different values of *Tg* for the same material can be observed depending on the experimental methods and the measurement conditions, e.g., different measurement frequencies or cooling rates. The glass transition temperature is assumed to be that of the onset of long-range segmental motion. Therefore, these differences can be attributed to the existence of various relaxation times of different motions of the macromolecular chains [10]. It has been also found [49] that, by using three independent experimental procedures (dielectric, thermally depolarized current, and calorimetric), the value of the glass transition and the value of the relaxation time at *Tg* can be correctly determined only if the thermal history is the same for all these experiments.

Dilatometry techniques are based on measuring the volume of an amorphous solid material as a function of temperature. The intersection of the straight lines V(T) estimates the value of *Tg* [51,64].

A commonly used method to determine *Tg* is differential scanning calorimetry (DSC). The glass transition on a DSC curve is visualized as a step-change in the heat flow curve [52,53]. This effect is a result of the change in heat capacity of the polymer at the transition range to the glassy state. It is important to note that the transition does not occur abruptly at one unique temperature but takes place over a range of temperatures. The glass transition temperature region is characterized by its onset, midpoint, inflection, and end set temperature. The midpoint temperature on the DSC cooling run is commonly accepted as an appropriate estimation of the glass temperature *Tg* [50,51,52,53,56].

Some authors [57,58] have suggested that different relaxations, related to changes in the content of heat, may appear during the DSC measured glass transition. Such relaxations associated with an increase in the mobility of the molecules can be observed as endothermic or exothermic changes in the heat flow in the region of the glass transition and can be attributed to the physical aging of glasses [5]. However, the determined *Tg* value strongly depends on the sample mass and the heating rate. The DSC method is dominant, although it is a tedious one [65]. Moreover, the study of the isothermal vitrification process that occurs during the isothermal curing of thermoset materials by conventional DSC involves time-consuming measurements of *Tg* as a function of curing time. Temperature modulated DSC (TMDSC) is a technique that enables the study of the vitrification process through measuring a sigmoidal change in the complex heat capacity, Cp*, from the value typical of liquids to that characteristic of glass [52]. The advantage of the TMDSC technique is improved resolution and sensitivity, as well as the ability to study the heterogeneity of the glass transition process.

Another technique for *Tg* determination is the thermal-mechanical analysis (TMA) technique, which involves determination of the coefficient of thermal expansion as a function of temperature. The *Tg* temperature is assumed as the onset point of the change in the coefficient of thermal expansion on the sample heating [51]. This method is more sensitive than DSC, but the determined *Tg* depends on the size and the surface roughness of the samples [65].

The most widely used method to measure the glass transition temperature of polymers is the dynamic mechanical (thermal) analysis (DMA/DMTA). This technique quantifies and records the response of a material to the applied oscillatory strain or stress as a function of temperature or frequency.

The glass transition appears as a change in the mobility of molecules in the glass transition region and can be detected in DMA data in three ways, i.e., as a sharp decrease in the storage modulus E′, as a peak in the loss modulus E″, and/or as a peak in the tan δ curve, respectively [10,59,60,65]. The changes in storage and loss modulus and the mechanical damping factor of a typical polymer at a particular frequency or temperature are related to various relaxation processes taking place in polymers. The main relaxation peak is known as α relaxation and is attributed to the glass transition.

Brostow et al. [10] have indicated that the results of *Tg* determined by the DMA method may also be different depending on whether the evaluation is made from the measurements of the loss modulus G″ or the tan δ = G″/G′. The glass transition temperature obtained from DMA is usually taken as the temperature of the maximum of the loss tangent [60].

In the electrical conductivity method, the resistance of the sample is plotted versus the inverse temperature and *Tg* is obtained from the intersection of two straight lines above and below the transition range. Large differences in *Tg* values are often found due to the wide temperature range of the transition region, reflecting different aspects of the same process. The factors that can influence the *Tg* value are mechanical inertia, thermal lag, thermal history of the samples, scanning rate, size of the specimen, and the laboratory apparatus [66]. It is worth emphasizing that the sensitivity of the DMTA method is approximately 1000 times greater than that of the DSC method [66].

Dielectric thermal analysis (DETA) describes the dielectric relaxations in amorphous systems in a way similar to that of the DMA technique [61]. A sharp increase in the permittivity and the dielectric loss peak are correlated with a glass transition [62]. According to McCrum et al. [63], such measurements allow the investigation of local and cooperative chain dynamics of polymers in an extremely wide range of frequencies (10^−2^–10^10^ Hz). In particular, segmental dynamics, usually referred to as relaxation α (in the low- and middle-frequency regions, up to 10^5^ Hz), may indicate dynamic glass transition [60].

The dielectric measurements reported by Uddin et al. [67] confirm the increase in the glass transition temperature of polyvinyl chloride (PVC) with the addition of barium titanate (BaTiO_3_, BT) filler. The dielectric constant (εr) measured at 40 °C increased from 7.6 for pure PVC to 16.1 with increasing BT content. It is suggested that BaTiO_3_ ceramic powder enhanced the dielectric properties of PVC.

The glass transition temperature *Tg* can also be determined by the optical method, which is simple and accurate for polymers with little thermal lag [65]. *Tg* is determined based on the temperature dependence of the refractive index. The glass transition process is associated with structural changes, which cause changes in the refracting index. This parameter is more sensitive to temperature change than the sample volume or modulus change used in the TMA method for *Tg* determination [65].

Another technique to investigate the glass transition in polymers is nuclear magnetic resonance (NMR). It provides valuable information that provides deeper insight into the properties of materials at the molecular level [68,69,70,71,72,73,74]. The NMR techniques make it possible to monitor the complex dynamics behavior of glassy polymers over a wide range of time scales by measuring different relaxation times, which dramatically change when the polymers undergo glass transition. The characteristic change in the relaxation time constants, as already mentioned, is attributed to the segmental motion of the polymer chain. Molecular dynamics in polymers cover a wide range of correlation times, from very fast processes on the order of pico and nano seconds up to slow motions on the order of milliseconds or even seconds. To cover this entire range, different techniques have to be applied. Using the NMR methods, “rapid” molecular motions, of the order of 10^−8^ s–10^−12^ s, can be extracted from the temperature dependence of the spin-lattice relaxation times T_1_, while the “slow” molecular motions of the order of 10^−6^–10^−2^ s can be obtained by the spin-lattice relaxation rate $T_{1\rho }$ or the off-resonance spin-lattice relaxation time $T_{1\rho }^{\mathrm{off}}$ in the rotating frame [75,76].

Irrespective of the method used, *Tg* may also be influenced by such features as the thermal history of the sample, as well as the conditions in which the experiments are performed. The glass transition is kinetic; thus, it is strongly influenced by the frequency (rate) of charging or deformation.

## 4. The Influence of Mineral Nanofillers on the Glass Transition of PVC

Nanofillers with particle sizes in the range of 1 to 100 nm are the modifiers frequently used nowadays in the technology of polymeric materials, applied mainly for the production of packaging films and rigid containers and automotive and industrial components. The reasons for the growing consumption of such fillers are the very good mechanical properties at low loadings, scratch resistance, superior barrier properties, enhanced fire-resistant properties, and improved heat distortion performance when compared to neat polymers.

Furthermore, nanofillers may also have a significant impact on changes in the glass transition temperature, which should be considered by planning specific applications of modified polymeric materials. In the case of PVC, the application of nanofillers usually leads to an increase in *Tg* when compared to that of the neat polymer; such an effect has been obtained by modification with zinc oxide (ZnO), titanium oxide (TiO_2_), calcium carbonate (CaCO_3_), halloysite nanotubes (HNTs), antimony trioxide (Sb_2_O_3_), and iron oxide (Fe_2_O_3_) [77,78,79,80,81,82,83,84]. An increase in the glass transition temperature, as measured by DSC, was found for PVC nanocomposites containing up to 20 wt.% of ZnO, prepared by the solvent casting method. This effect is probably related to the strong interaction between ZnO nanoparticles and the PVC matrix, owing to a large amount of rigid amorphous fraction in the amorphous region [77].

According to one reference [78], a higher glass transition temperature of the PVC nanocomposite film, containing up to 6 wt.% of TiO_2_ prepared by the same method as in another paper [77], clearly indicates the appropriate diffusion of nanofillers that resonate with the great interfacial surface region among inferiorly mobilized chain segments. The growth of *Tg*, observed by the DMA measurements, depends on the loading of nanoparticles; usually, an increase is observed up to the saturation level of about 6%, followed by a decrease with a higher concentration of nanoadditive particles, inducing the formation of agglomerates, thus lowering the plastification effect of the PVC matrix. Based on the DMA measurements of PVC nanocomposites with TiO_2_, Fe_2_O_3_, and ZnO, Sadek et al. have confirmed that the observed increase in the *Tg* values in comparison with those of unfilled PVC may be explained by an increase in the stiffness of PVC, which restricts its chain mobility. In this case, the saturation level was found at about 10% of loading [83].

A similar temperature effect was found for PVC modified by uniformly distributed CaCO_3_ nanoparticles in concentrations up to 5 wt.%, introduced into the PVC matrix during in situ polymerization. The glass transition temperature of the PVC was slightly shifted towards higher values by the simultaneous influence of the nanoparticles on the restriction of segmental and long-range chain mobility (about 1.1–1.4 °C) compared to neat PVC [79].

Xiong et al. have reported an increase in the glass transition temperature of the PVC/CaCO_3_ composite obtained by the introduction of CaCO_3_ particles during the reaction of Ca(OH)_2_ with CO_2_ inside the cavities of microporous PVC with the pore size in the range between 0.2 and 2 µm [80]. Based on SEM and TEM observations, it was found that the in situ produced CaCO_3_ nanoparticles, with a size below 50 nm, are uniformly dispersed in the PVC matrix. On the basis of the XRD patterns, it was suggested that pseudo-amorphous crystals and defect-rich crystals are formed. Moreover, DMA data indicate a higher *Tg* of the in situ PVC/CaCO_3_ nanocomposites when compared with those of the common PVC/CaCO_3_ nanocomposites.

The impact of different processing conditions and contents of plasticizers on the glass transition of PVC compounds containing 50 phr (parts per hundred resin) of CaCO_3_ was determined on the basis of DSC and DMA measurements by Liu et al. [84]. Depending on the procedure of the introduction of CaCO_3_ powder into the PVC compound with the same plasticizer content, different values of *Tg* were found. The addition of CaCO_3_ powder by a one-step procedure results in singular *Tg* values, while, when using a two-step procedure, two separate *Tg* values were detected. It should be added that these values determined by DSC and DMA were different and the difference between them is of about 40 °C or more.

A shift in the *Tg* value towards a higher temperature for PVC/ PMMA-grafted HNTs nanocomposites, as determined by DSC, indicating changes in polymer thermodynamics, has been reported by C. Liu et al. Furthermore, the PMMA shell on halloysite particles increases the interaction between this filler and the PVC matrix, restraining the thermal motion of the PVC chains, leading to an enhancement of the thermal stability of the PVC [81].

A restrictive impact on the thermal motions of PVC molecular chains, leading to an increase in *Tg*, has been identified by Xie X.-L. et al. The experiments were executed by DSC and DMA for PVC with nano-sized antimony trioxide (Sb_2_O_3_) particles modified by in situ methyl methacrylate (MMA)/Sb_2_O_3_ polymerization [82]; an effect similar to that described above [81] has been observed for HNT modification. The formation of the PMMA shell on nano-Sb_2_O_3_ particles increases the interaction between the nanofillers and the PVC macromolecules. The nanofillers restrain the thermal motion of PVC molecular chains by adding 5.0 wt.% of in situ PMMA modified nano-Sb_2_O_3_ particles, leading to an increase in the *Tg* of PVC from 69.74 °C to 74.33 °C. However, it was observed that an increase in filler concentration up to 7.5% led to an agglomeration of particles and weakening of the interactions between nano-Sb_2_O_3_ particles and PVC, and, consequently, to the lowering of the PVC *Tg* to 71.63 °C [82].

It has been found [85] that differences in the *Tg* values of unfilled PVC, the composites with 3 wt.% of organically modified bentonite and hectorite clays as well as with 2 wt.% of talc, calcium carbonate, and kaolin, are insignificant, indicating that there are no substantial changes in the polymer thermodynamics. The *Tg* values within the range from 74 °C to 77 °C are comparable to that of unfilled PVC; the methods of obtaining the composites, i.e., a direct mixing in the molten state and a pre-gel method, did not influence this value either.

The effect of using montmorillonite (MMT) as nanoadditives for the PVC has been described in the literature, indicating a fairly different impact of this filler on glass transition [86,87,88,89,90].

Wan et al. [87] have found that the glass transition temperature measured by the DMTA of intercalated PVC nanocomposites with sodium montmorillonite (Na^+^-MMT) and two organic MMTs, modified with trimethyloctadecyl ammonium (MMT-C18) and dimethyldioctadecyl ammonium (MMT-2C18), was shifted to a higher temperature range compared to that of pristine PVC. This effect was explained by the restriction of polymer chain mobility within the interlayer. It has been noted that, for the same MMT loading, the *Tg* of PVC/Na^+^-MMT composites is slightly lower than those of PVC/organic MMT composites because the intercalation extent between the PVC chains and Na^+^-MMT layers is not as high as those of PVC/organic MMT composites, and, consequently, the PVC segments have higher mobility in PVC/Na^+^-MMT composites than those in the PVC/organic MMT composites.

Xu at al. have reported that the glass transition temperature of PVC/organic-montmorillonite composites (Org-MMT) prepared by intercalation in a molten state is slightly lower than that of virgin PVC [86]. The interlayers of MMT may play the role of a plasticizer, increasing the distance between PVC chains and thus lowering the interaction forces between PVC molecules, consequently leading to a lower glass transition temperature of PVC/Org-MMT. For the materials containing above 5 phr of nanofillers, the exfoliated structures—with physical junctions between PVC blocks and clay plates—dominate, leading to certain limitations of PVC segment movement and, thus, to an increase in DSC determining the glass transition temperature of PVC.

The lowering in the *Tg* in comparison to pristine PVC, related to introduction into the polymer from 2 to 10 wt.% of Na^+^-montmorillonite modified with cetyl ammonium bromide (organo-MMT), was confirmed on the basis of DSC analysis by Sudhakar et al. [89]. Accordingly, this phenomenon may be explained by a reduction in the intramolecular interactions in the polymer in the presence of entrapped exfoliated organo-MMT layers.

A different effect has been found for nanocomposites synthesized via in situ intercalative PVC polymerization with organophilic montmorillonite (OMMT) [88]. The DSC thermograms of pure PVC and the PVC/OMMT systems containing 1, 3, and 5 wt.% of OMMT, respectively, indicate a gradual slight increase in the *Tg* for PVC/MMT nanocomposites compared with that of pure PVC (85.3 °C) until 87.6 °C for 5 wt.% of MMT. According to these authors [88], it may be assumed that some PVC chains in the nanocomposites are immobilized inside and/or onto the layered clay, which prevents the segmental motion of the polymer chains.

The thermal effects of dioctyl phthalate on the properties of PVC/OMMT nanocomposites have been reported by Chen et al. [90]. On the basis of the DSC curves, a significant decrease in the *Tg* of PVC/OMMT/DOP nanocomposite with DOP loading increase was noticed. The *Tg* value of rigid PVC/OMMT nanocomposite is around 80 °C, but, for the nanocomposite containing 50 phr of DOP and the same content of OMMT, it is around 0 °C.

## 5. The Influence of Oligomeric Silsesquioxanes on the Glass Transition of PVC

In many cases, it has been found that the use of polyhedral oligomeric silsesquioxanes (POSS) as nanofillers, irrespective of the functional groups (POSS) [14,15,91,92,93,94,95,96] attached to the POSS cage, leads to the plasticizing effect of PVC, i.e., to a lowering of *Tg*. The influence of 10, 15, and 20 wt.% methacryl polyhedral oligomeric silsesquioxanes (POSS) on the *Tg* of PVC was described by Soong et al. [91], who observed a monotonous decrease in the α-transition temperature and related changes in mechanical properties (lowering of storage modulus) as a clear indication of the plasticizing role of POSS on the PVC matrix. This effect, observed for the materials containing up to 15 wt.% of POSS, may also indicate an increase in the PVC free volume caused by the addition of POSS molecules.

**Figure 2:**
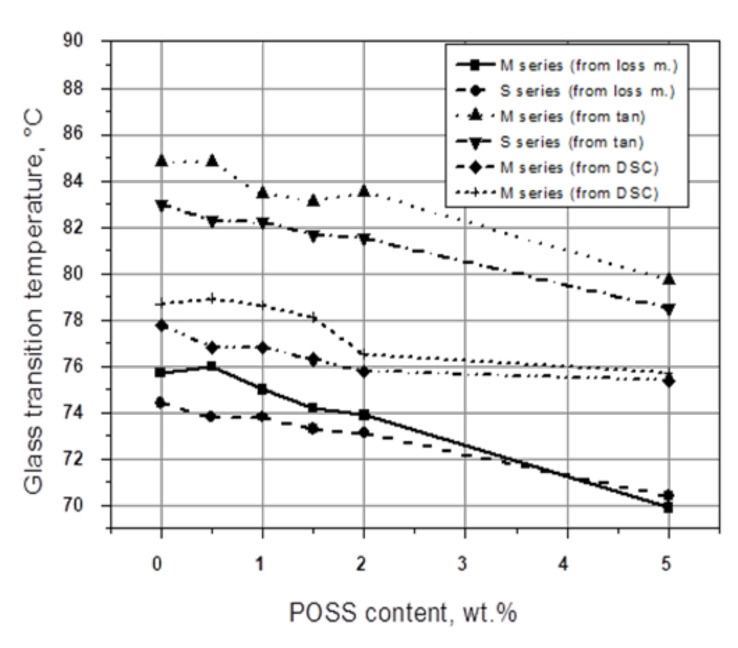
The *Tg* values from the DSC thermograms, loss modulus, and tangent δ DMTA spectra for PVC/POSS composites as a function of POSS concentration [14].

A certain lowering of *Tg* was found in our studies for PVC modified with POSS containing various functional groups [14,15], measured by DSC and DMTA, at the frequencies f = 1.0 and f = 10.0 Hz, respectively, where the position of tan δ at its maximum, as well as the maximum value of G″, were taken as the *Tg* region. For PVC modified with POSS containing 3-chloropropyl groups (CP-POSS), in a concentration between 0.5 and 5 wt.%, a decrease in the *Tg* with increasing modifier content was also found. This effect was independent of the method of POSS incorporation, i.e., direct solid powders mixing (M series) or the addition of POSS to the PVC solution in THF (S series). Moreover, the real values of *Tg*, obtained using all three methods, DSC, and DMTA (from G″ and tan δ), vary substantially; a difference of about 10 °C between the *Tg* values for every POSS concentration may be noted. The DSC determined *Tg* values are located between the G″ and tan δ evaluated from the DMTA related curves, measured at a charging frequency of 1.0 Hz (Figure 2). The differences between the *Tg* values measured by DMA and DSC seem to be affected by the dissimilar stimulation of macromolecular chain mobility (DSC versus DMTA) and different response times of the chain motions (tan δ versus G″).

**Figure 3:**
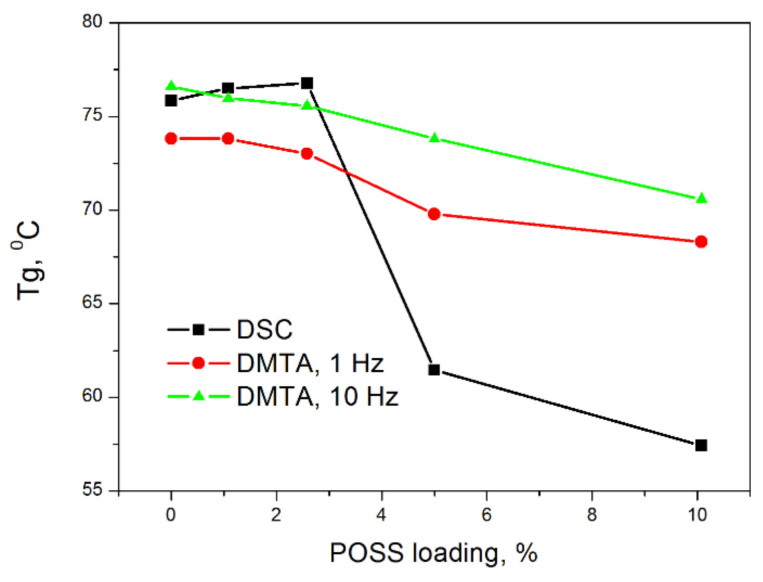
The changes in *Tg* of PVC nanocomposites as a function of MeOctPOSS concentration measured by DSC and DMA at various frequencies [15].

A similar plasticizing effect on PVC appears as a result of the addition of POSS with methacryl and octyl groups attached to the silsesquioxane cage (MeOct-POSS) [15]. As follows from Figure 3, for a POSS concentration of 5 and 10 wt.%, a noticeable lowering in DSC determined *Tg* was observed. In this case, a decrease of *Tg* from 75.9 °C for the neat PVC sample to 57.4° C for PVC modified with 10 wt.% of MeOctPOSS, was identified. In the case of DMA measurements, it may be seen that, the higher the measurement frequency, the weaker the *Tg* dependence on POSS concentration, signifying the leading effect of the rate of charging/discharging on the macromolecular chain mobility.

The tendency of MeOct-POSS to plasticize the PVC matrix is most likely a result of the integration of relatively long POSS octyl groups among the PVC macromolecules, influencing the intermolecular distance and thus the temperature-dependent chain mobility.

To sum up, we have found that both CP-POSS and MeOct-POSS may be used as non-phthalate plasticizers of polyvinyl chloride [14,15]. Moreover, irrespective of the type of POSS nanomodifier, the higher the measurement frequency, the higher the value of *Tg*. A similar tendency was found for PVC/CNT composites [13], which will be explained in the following part of the paper.

The lowering of the glass transition temperature to near room one may be achieved if ternary blends of PVC/DOP/POSS with methacrylic groups are formed, leading to a polymeric material with ductile behavior [93]. The ability of methacryl-POSS to plasticize PVC has been confirmed since it is much less volatile due to its hybrid organic-inorganic structure when compared to DOP.

The influence of methyloacrylpropyl POSS (MAP-POSS) on the plastification of PVC/chlorinated polyethylene blends (PVC/CPE) has been demonstrated in one study [94], showing that the addition of MAP-POSS leads to a reduction in the *Tg* of the PVC/CPE binary blend from 76 °C to about 70 °C, simultaneously with an increase in the storage modulus and a decrease in the tan δ, similar to the results published by Soong et al. [91]. It has been suggested that the MAP-POSS molecules may be located in the free volume between PVC and CPE molecule chains and thus may originate a plasticizing effect at the molecular level.

A substantial plastification of the PVC matrix has been reported in one reference [92], as reached by the addition of fairly high amounts of poly(ethylene glycol)-polyhedral oligomeric silsesquioxane with a weight fraction from 0.2 to 0.5. A decrease of the *Tg* from 72.0 °C for pure PVC to 44.3 °C by the weight fraction of 0.5 of PEG-POSS has been linked to the formation of hydrogen bonds between the PEG parts of PEG-POSS and the PVC matrix. Furthermore, the fairly high flexibility of PEG may contribute to the reduction in the glass transition temperature of PVC. Moreover, the predominant bulk nature of POSS acting with separate PVC molecules may increase its free volume and thus may result in the lowering of the *Tg*. The DMA results confirmed that the decrease in the *Tg* value of plasticized PVC modified by chlorobenzylethylisobutyl-POSS can also be explained by an increase in the free volume of the PVC/POSS nanocomposites due to an increase in POSS content, suggesting that the nanoparticles may act as plasticizers in this case [95].

The opposite effect, i.e., an increase in *Tg*, has been noted for PVC modified with polyhedral oligomeric silsesquioxane containing 3-chloropropyl groups (CP-POSS) in concentrations between 3 and 11 phr [96]. The slight increase in *Tg*, from 70.7 °C (for a pure PVC) to 73.3 °C for the sample containing 3 phr of POSS, has been attributed to molecular interaction between the nano-size CP-POSS molecules and PVC chains. A relative lowering of the *Tg* to the value of around 72 °C for higher concentrations was explained by the existence of multi-polar groups in CP-POSS characterized by a lower melting point, with a suggestion of a slight plasticizing effect on PVC.

## 6. The Influence of Carbonaceous Nanofillers on the Glass Transition of PVC

Carbonaceous nanofillers, such as graphene (GN) and carbon nanotubes (CNTs), play a promising role in the modification of PVC, also affecting the properties in its vitreous state [13,97,98,99,100,101,102,103,104,105,106,107,108,109,110,111]. Due to the nano-sizes of these additives, a direct impact on the macromolecular chains seems possible. It would lead to an improvement in the physical properties, such as an increase in mechanical properties, electrical conductivity, environmental resistivity, etc. An important remark is that the influence of carbonaceous materials is strongly dependent on the nanofillers’ content in the polymeric matrix. The modified properties are different in the conditions below and above the percolation threshold [112]; this effect concerns the mechanical, electrical, and rheological properties. Another very important task is to develop technological knowledge with regard to assembling nanocomposites with a high level of homogeneity, a topic widely discussed in the literature [97,98,99,100,101,102,103,104,105,106,107,108,109,110,111,112,113,114,115].

The studies of the effect of carbon nanotubes on the glass transition of PVC nanocomposites have been shown to be of top interest as far as the thermal properties of PVC nanocomposites are concerned [13,99,100,101,102,103,104,109].

The role of multi-walled carbon nanotubes (MWCNTs) on the alteration of the glass transition of PVC, determined by DSC, DMTA (by 1.0 and 10.0 Hz), and electrical loss factor (by 1000 Hz), has been widely studied [13], and a considerable influence of both measurement frequency and CNT content on the glass transition temperature of PVC was ascertained.

**Figure 4:**
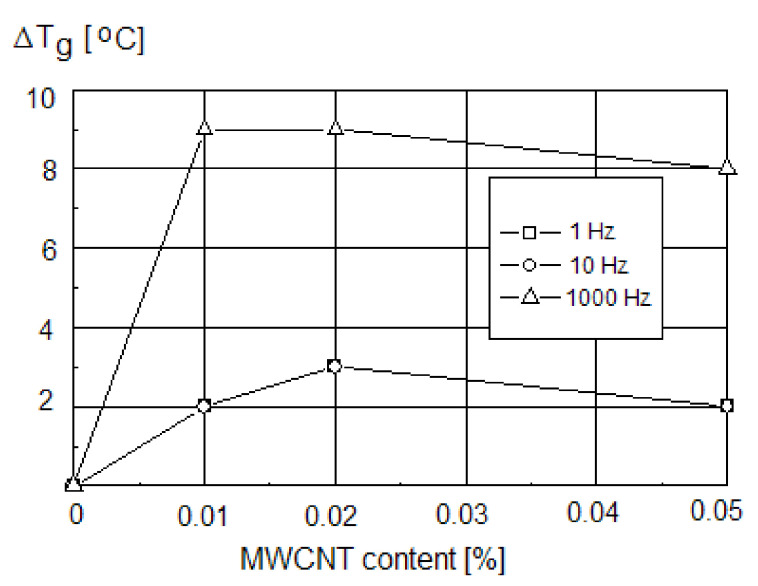
The changes in *Tg* of PVC nanocomposites at different measurement frequencies as a function of CNT content [13].

An increase in the glass transition temperature with the content of MWCNTs increasing from 0.01 wt.% to 0.05 wt.% was noted, whereas the impact of MWCNT on the *Tg* value measured by the DSC was less evident (an increase of *Tg* from 69 °C for neat PVC to 70.5 °C for PVC with 0.05% of MWCNT). *Tg* changes could be observed even for a very low MWCNT concentration, indicating the effect of nanotubes on the PVC chain mobility. The saturation-like effect was observed for the MWCNTs content of 0.01–0.02 wt.%. Regarding the influence of the measurement frequency, a shift of *Tg* for PVC/CNT nanocomposite of about 3 °C at the frequencies f = 1.0 Hz and f = 10.0 Hz, and of 9 °C at f = 1000 Hz (Figure 4), was observed [13].

Comparing the DMTA, dielectric, and DSC measurements, the greatest influence on the determination of the *Tg* of PVC/MWCNT nanocomposites seems to be brought about by the periodically altering charging of the samples. The effect is probably due to the different frequency-dependent responses of the nanotubes’ movement by temperature-reliant PVC chain mobility.

**Figure 5:**
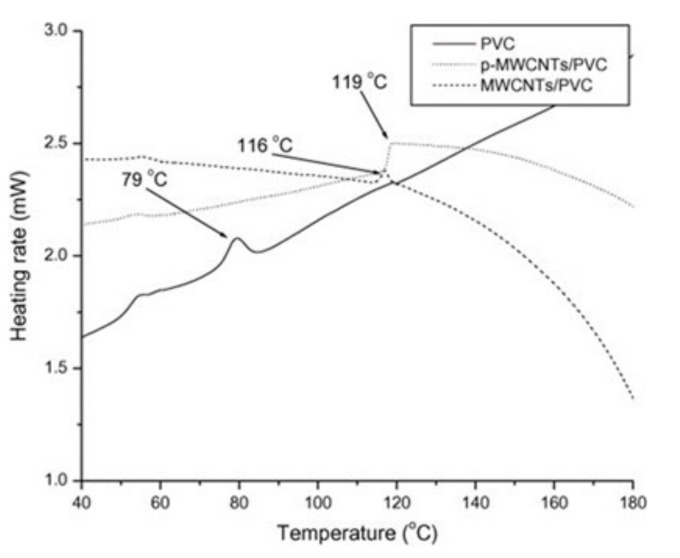
The *Tg* values on the DSC thermogram of the cooling run of PVC and PVC/MWCNT nanocomposites [101].

Mkhabela et al. [101] have reported a significant influence of the chemical treatment of nanotubes on the *Tg* of PVC nanocomposites. The addition of 0.5 wt.% of phosphorylated MWNCT (*p*-MWCNT) resulted in a shift of the glass transition temperature towards a higher value (119 °C) compared with that of the unmodified MWCNT composite (116 °C) and with pristine PVC (79 °C) (Figure 5), which may be explained by more evident interactions between chemically modified nanoparticles and polymer macromolecules.

An increase in the glass transition temperature for nanocomposites with a segregated network structure, determined by DSC and TMA, was described by Mamunya et al. [102]. Multiple effects were noted, such as an increase in the *Tg* and simultaneous flow temperature Tp, due to the physical interaction between carbon nanotubes and the polymer matrix, and the presence of a new phase (boundary layer) between the polymer macromolecules and the surface of the nanotubes. These boundary layers exhibit different properties than those of non-modified PVC, also leading to restrictions of macromolecular mobility.

The investigations of PVC modified by nucleophilic substitution to introduce hydroxyl pendant groups, capable of forming ester linkages with carboxylic groups on MWCNTs, allowed proving that the *Tg* of PVC may be related not only to the regularity of the structure of macromolecules (tacticity) but also to interactions between nanotubes and the PVC macromolecules, as reported Salavagione et al. [103]. The chemically modified PVC (mPVC) with an increasing grade of substitutions was characterized by a higher content of syndio and a lower content of isotactic triads. It was found that the higher the degree of substitution, the higher the *Tg* value determined as a maximum of tan δ. It was found that the influence of MWCNT on glass transition was most evident for PVC samples with the lowest degree of substitution; the value of *Tg* of mPVC/MWCNT was, in this case, about 5 °C higher when compared with the *Tg* of mPVC. These authors [103] suggested that the reason for an increase in the *Tg* value of the nanocomposite matrix was local conformational changes in the PVC macromolecules due to the presence of CNTs occupying free volumes close to the isotactic sequences of PVC chains, which increased the density of packing and thus affected the restriction of the segments’ mobility. The effect of MWCNT on *Tg* for mPVC samples, characterized by a higher degree of substitution, was less evident, and the *Tg* values within the temperature range between 98.7 °C and 100.6 °C were similar to those in mPVC.

In another work, Salavagione et al. have described the influence of 5 wt.% of ester-functionalized MWCNTs on the thermal properties of the nanocomposite with PVC synthesized in different conditions, resulting in a diversity of contents of syndio and isotactic triads [104]. Similarly, like in one reference [103], the authors have found that, in both the unmodified PVC samples and in the nanocomposites, the glass transition temperature depends on the tacticity of the polymer, i.e., *Tg* increases as the isotacticity decreases. Additionally, due to the addition of 5 wt.% of MWCNT, the higher the content of the isotactic sequences in the pure polymer, the greater the *Tg* shift towards higher temperatures. This effect indicates significant interaction between nanotubes and the sequences with a higher regularity of structure.

To improve the homogeneity of dispersion of the nanotubes in the PVC matrix, poly(*n*-butyl methacrylate) (PBMA) was grafted onto multi-walled carbon nanotubes (PBMA-g-MWCNTs) using atom transfer radical polymerization [99]. The incorporation of 0.2 wt.% of pristine MWCNTs resulted in a slight increase in the glass transition temperature of PVC taken as the tan δ peak. However, the effect of shifting *Tg* towards higher temperatures was more pronounced after the addition of PBMA-g-MWCNTs in a concentration between 0.1 and 0.5 wt.%.

A similar tendency of temperature increase has been observed as a result of introduction of double-C60-end-capped PBMA in PVC; however, in this case, the *Tg* value is inferior, i.e., 63 °C, due to the lower drying temperature of 40 °C, which might not be sufficient to remove the residual casting solvent [100].

The influence of PVC molecular weight and CNTs concentration in porous films prepared by the nonsolvent-induced phase separation method has been reported by Molla-Abbasi [109]. The DSC results showed that the *Tg* values of both PVC grades differing in molecular weight containing 2 wt.% of CNTs were higher compared with that of PVC pristine samples due to the limiting effect of CNTs on the segmental motions of the polymer. Therefore, this raise was more pronounced for the PVC with a lower molecular weight. A further increment in *Tg* value was observed when 40 vol% of ethanol as nonsolvent was added to the system, which proves the associating ability of CNTs with polymer chains during the phase separation process [109].

According to Aljaafari et al. [98], the addition of carbon nanopowder (CP) and, separately, carbon nanotubes in a concentration up to 15 wt.% leads to the measurement frequency-dependent changes in the PVC glass transition temperature. A slight variation in the *Tg*, together with the simultaneous broadening of tan δ peak and a lowering of its intensity, the effect practically independent of the loading of either nanofillers, indicates an interaction between the fillers’ surfaces and the PVC chains, imposing a constraint on their mobility. A strong physical interaction between carbon filler and PVC chains confirmed the DSC results obtained for the PVC/carbon fiber nanocomposites (PVC/CF) reported by Pakdemir [116]. According to him, the higher *Tg* value of PVC/CF (86.7 °C) composite in comparison with the *Tg* of pure PVC (76.8 °C) indicates that physical interaction of the polymer with the CF surface reduces the chain mobility and free volume, which results in an increase in *Tg*.

The broadening of tan δ peaks and the shift of the maximum have also been observed for PVC modified with graphene, which was probably related to the restriction of segmental relaxation of the PVC macromolecules together with the reinforcing effect of GN and a reduction in chain segmental mobility, for the weight fraction of GN between 1 and 3 wt.% [96]. Similar observations have been reported by Aljaafari et al. [98] for the PVC nanocomposites containing carbon additives, such as CNT and CN fillers.

Wang et al. [105] have reported that a low loading (0.36–1.08 wt.%) of multilayer graphene (MLG) may weaken the intermolecular interactions and improve the segmental motion of PVC chains, finally resulting in a slightly lower *Tg* and MLG/PVC when compared to that of the neat PVC. According to these authors [105], when the MLG concentration increases to 1.8 wt.% in the polymer matrix, its particles begin to squeeze each other, consequently hindering the motion of PVC chains, resulting in higher *Tg* values, although these changes are very small, lower than 1 °C.

**Figure 6:**
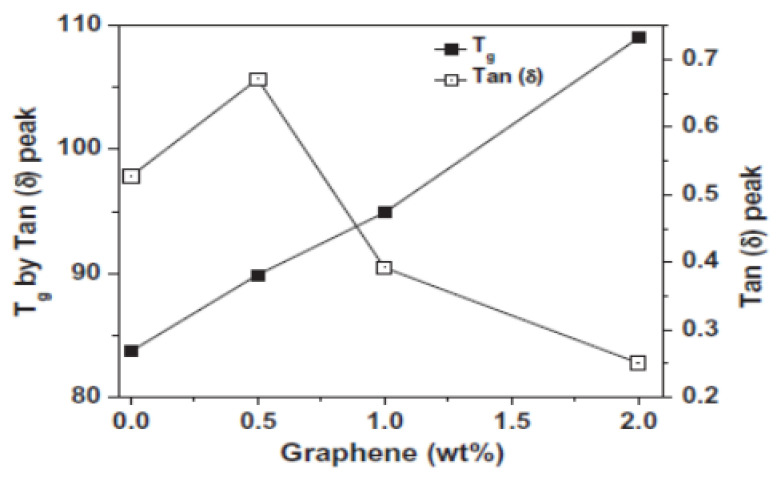
The *Tg* and the tan δ values vs. weight fractions of graphene in the PVC matrix [106].

Vadukumpully et al. [106] have found that molecular dynamics in PVC composites are influenced by sonicated, well-dispersed graphene nanoflakes in concentrations up to 2 wt.%. With increasing graphene content, an increase in *Tg* was observed, accompanied by a significant reduction in the loss factor (Figure 6), which is probably related to the presence of graphene layers acting as a “physical crosslinks”. Some authors have identified a difference in *Tg* values, as obtained by DMA and DSC tests, which may be explained by the test frequency dependent *Tg* value. This conclusion is in agreement with our studies on the modification of PVC with CNT and POSS containing various functional groups [13,14,15].

A restriction in the chain mobility induced by the graphene layers (GN) in concentrations in the PVC matrix up to 3wt.%, followed by a significant augmentation of the *Tg*, was confirmed for the nanocomposite fibers [107]. The *Tg* value of the composites was estimated from the DSC thermograms, showing a characteristic inflection point in the temperature range 77–94 °C for all PVC/GN fibers; the *Tg* of the sample containing 3 wt.% GN was by about 6 °C higher than that of pure PVC. Mindivan F. et al. have reported that the increase in *Tg* in PVC composites containing from 0.1 wt.% up to 1.0 wt.% of graphene nanoplatelets (GNP) indicates a restriction in segmental relaxation. However, due to the formation of hydrogen bonds between GNP and the polymer, the *Tg* values of all nanocomposites (between 34.99 °C and 44.36 °C) were lower than that of the neat PVC (44.71 °C) [110].

The influence of carbonaceous fillers on the glass transition temperature region was also observed in the plasticized PVC blends modified with GN [111] and RGO [117]. The introduction of 0.06 wt.% to 2.0 wt.% of graphene to the mixture with 20 wt.% of the plasticizer caused only a slight increase in the *Tg* in comparison with that of pristine PVC (about 1 °C), while no significant dependence of this temperature on GN concentration or method of preparation of composites in the presence of DOP was observed, i.e., by high-energy ball milling and by conventional stirring [111]. For the compounds containing 40 phr of the plasticizer, the influence of RGO on this temperature by classical DSC was difficult to unequivocally determine in view of a very wide *Tg* range and very low heat flow steps in this range. Therefore, the procedure of inducing physical aging in the amorphous phase was used, and an increase in the *Tg* from 7 °C to 10 °C with increasing loading of RGO in the PVC matrix from 0.5 wt.% to 5.0 wt.% was observed. This effect is due to considerable constraints of molecular relaxation in the amorphous phase, inducing an increase in *Tg* [117].

Salavagione et al. [108] have published the results of research on nanocomposites of PVC with reduced graphene oxide (RGO), isocyanates-modified reduced graphene oxide (iRGO), and RGO grafted on PVC, modified with nucleophilic substitution PVC (RGO-e-PVC). They have found that the *Tg* values determined by DSC do not depend on the RGO and iRGO content, which may suggest that there are no molecular interactions between the RGO and PVC chains. The PVC/RGO and PVC/iRGO samples behave as physical mixtures, and their segmental mobility is the same as in the pristine PVC. In contrast, a shift of about 20 °C of *Tg* towards higher temperatures, when compared to that of pristine PVC, was noted for the RGO-e-PVC samples. This effect was related to strong interactions between the plates of RGO and the contiguous polymer segments, limiting the mobility of chains in the zones of higher free volume, corresponding to a higher content of isotactic sequences, as in the case of MWCNTs [103]. However, the increase in *Tg* is greater when compared to the rise in *Tg* found for PVC-grafted MWNT characterized by the same degree of modification, where the value of *Tg* was shifted towards higher temperatures by about 5 °C relative to that of PVC of a similar composition (∼1.3 wt.% MWNTs and 1.2 wt.% RGO) [103]. The different effects were explained not only by the higher aspect ratio of graphene but also by the potential occupation of the space of the large free volume, attributed to the conformation of sufficiently long sequences of GO platelets, which increase the packing density [108].

The effects of the addition of carbonaceous nanofillers on the glass transition temperature as a function of the filler content, sizes of its molecules, and the method of composite preparation are presented in Table 1 after [13,97,98,99,101,102,103,104,105,106,107,108,109,110,111,116,117]. It may be concluded that the addition of carbonaceous nanofillers leads to an increase in the *Tg* of PVC, although this increase is strongly dependent on a variety of factors, such as the length and diameter of the CNTs, the chemical functionalization of CNTs and GN, as well as the interaction of the fillers and the matrix. Moreover, the type and properties of PVC and the homogeneous distribution of carbonaceous nanofillers in the PVC matrix also significantly contribute to the glass transition. As follows from Table 1, different *Tg* values are usually observed if different investigation methods are applied.

## 7. Conclusions

The glass transition temperature, *Tg*, is a very important property of glass-forming materials, determining their industrial application and the types of processing of these materials. Polyvinyl chloride belongs to the most frequently used materials in the production of nanocomposites because of its beneficial properties, such as its comparatively low cost, extensively developed processing, the possibilities of modification of its mechanical properties, and its high environmental resistance. The restrictions of PVC applications are mostly related to the relatively high glass transition temperature of this polymer, resulting from strong polar interactions between chlorine and carbon.

This work presents a comprehensive overview of the influence of the nanoadditives most commonly used in PVC technology, including mineral nanofillers, oligomeric silsesquioxanes, and carbonaceous nanofillers.

The influence of nanoadditives on PVC glass transition are well established experimentally, as demonstrated in this review. These effects are accounted for by changes in the chain mobility resulting from increased intermolecular distances due to the plastification and/or from the additive induced rising of macromolecular stiffness. For the chemical interactions between the functionalized additives and macromolecular chains, similar effects may be observed.

Most of the research data presented in this review have shown that the modification of PVC with nanofillers, such as mineral and carbonaceous nanofillers, contributed to the increase in *Tg*, which can be attributed to the restriction of macromolecular mobility.

The lowering in the *Tg* is mainly associated with the introduction of POSS nanoadditives, providing an interaction between the nanoadditives and PVC chains, where the *Tg* lowering originates from the plastification effect.

As far as the effects of carbonaceous nanofillers addition are concerned, their influence on the *Tg* depends on a variety of factors, including the content, size, and aspect ratio of the fillers, chemical functionalization, and methods of composite production, as well as on the structure of the PVC used as a matrix of the composites.

It should be stressed that the experimental technique may significantly influence the value of *Tg*. Nevertheless, we sincerely hope that this critical review may provide a guide to researchers who intend to study the effects of the addition of nanofillers to a PVC matrix on its glass transition region.

## Figures and Tables

**Table 1:** Summary of the reported changes in *Tg* of PVC with carbonaceous nanofillers.

Reference	PVC Type/wt.% of Carbonaceous Filler	Processing	Testing Method	*Tg* Value
Sterzynski T. et al., 2010 [13]	PVC powder S–70, Anwil S.A., Wloclawek, Poland	Solution casting from THF followed by kneading	DMA tan δ	frequency-dependent 65 °C (1.0 Hz)–71 °C (10.0 Hz)
			DSC	69.0 °C
			Dielectric tanδ dl	85.0 °C
	0.01, 0.02, and 0.05 wt.% of MWCNTs (average diameter of about 10 nm), Nanocyl S.A., Sauberville, Belgium)		DMA tan *δ*	frequency and MWCNT content-dependent 67.0–68.0 °C (1.0 Hz) 73.0–74.0 °C (10.0 Hz)
			DSC	69.4–70.5 °C
			Dielectric tan δdl	93.0–94.0 °C
Hasan M. and Lee M., 2014 [97]	PVC powder, (M = 1020), Yakuri Pure Chemicals, Kyoto, Japan	Solution casting from THF	DMA tan δ	PVC no data
	1–3 wt.% GN (thickness ~8 nm, length ~500 nm), Iljin NanoTech, Seoul, Korea			GN content-dependent 52.1 °C (PVC/1% GN)–52.9 °C (PVC/3% GN)
Aljaafari A. et al., 2007 [98]	PVC (powder fraction average 100 µm, ρ = 1.37 g/cm^3^), Sabic Company, Riyadh, Saudi Arabia	Solution casting from THF followed by compression molding	DMA tan δ	frequency-dependent 89.0 °C (1 Hz)–97.1 °C (50 Hz)
	0.5, 1, 2, 5, 10, 15 wt.% of CNTs (diameters from 12.7 to 24.4 nm), Center of Ecxellence in Nanotechnology, KFUPM, Saudi Arabia			87.00–91.00 °C (1 Hz) 90.00–92.00 °C (5 Hz) 96.11–97.12 °C (50 Hz) no dependence on concentration
	1, 2, 5, 10, 15 wt.% of carbon nanopowder (CNP) (average diameter below 50nm), Sigma Aldrich, Darmstadt, Germany			86.00–87.96 °C (1 Hz)90.00–91.01 °C (5 Hz)96.12–97.11 °C (50 Hz)no dependence onconcentration
Shi J.-H. et al., 2007 [99]	PVC (Mn = 60,000 and Mw = 106,000), Sigma Aldrich, Darmstadt, Germany	Solution casting from THF	DMA tan δ	85 °C
	0.1,0.2 and 0.5 wt.% of PBMA-g-MWNTs (diameter within 20–30 nm), synthesized by the cited authors			~86–~90 °C no dependence on concentration
	0.2 wt.% of pristine MWCNT (diameter within 20–30 nm), Shenzhen Nanotech Port Co. Ltd, Guangdong, China			~86 °C
Mkhabela V. J. et al., 2011 [101]	PVC, no data	Kneading-extrusion	DSC	79 °C
	0.5 wt.% pristine MWCNT 0.5 wt.% phosphorylated-MWCNT (*p*-MWCNT), synthesized by the cited authors			116 °C (PVC/MWCNT) 119 °C (PVC/*p*-MWCNT)
Mamunya Y. p. et al., 2010 [102]	PVC C–7058 M (powder form, particles 100 µm, *ρ* = 1.37 g/cm^3^, Oriana company, Kalush, Ukraine	Dispersion of MWCNT in ethanol solution- homogenization with PVC by grinding—compression molding	DSC, TMA	87 °C (DSC), 84.1 °C (TMA)
	0.08, 0.10, 0.33, 0.47 wt.% of MWCNT (diameter within 12–20 nm) TMS-petsmash Kyiv, Ukraine		DSC	85–87 °C no dependence on concentration
			TMA	84.6–85.4 °C no dependence on concentration
Salavagione H. J. et al., 2010 [103]	Laboratory synthesized PVC and PVC modified by nucleophilic substitution (mPVC) with various degree of substitution	Esterification reaction MWNTs-mPVC-precipitation	DSC	87.4–101 °C degree of PVC substitution-dependent
	1.3% units of functionalized MWNTs-mPVC (average outside diameter of 13 nm), Bayer, Leverkusen, Germany			92–106 °C degree of PVC substitution-dependent
Salavagione H. J. et al., 2012 [104]	PVC (bulk polymerization at 70 °C, Mn = 44,000), Atochem, SpainPVCs synthesized in various conditions (Mn = 21,500 and 54,300)	Solution casting	DSC	78.1–91.5 °C PVC grade-dependent
	5% of ester-functionalized MWCNTs (average diameter of 13 nm), Bayer, Leverkusen, Germany			82–90.5 °C PVC grade-dependent
Wang H. et al., 2017 [105]	PVC powder type SG–5, Sichuan Jinlu Group, Deyang, China	Kneading-roll-milling-compression molding	DMA tan δ	93.11 °C
	0.36, 0.72, 1.08, and 1.80 wt.% of multilayer graphene (MLG) (layer number less than 10 graphitic layers, lateral size is as large as 10–15 µm and thickness about 1–3 nm), Sichuan Jinlu Group, Deyang, China			MLG content-dependent 92.76 °C (PVC/0.78% MLG) 95.58 °C (PVC/1.80% MLG)
Vadukumpully S., 2011 [106]	PVC powder (Mw = 120,000), Sigma-Aldrich, Darmstadt, Germany	Solution casting from DMF	DMA tan δ	84 °C
	0.5, 1, 2 wt.% of GN nanoflakes prepared from graphite (thickness ~1.18 nm, corresponding to 1–3 graphene layers), synthesized by the cited authors			GN content-dependent 90 °C (PVC/0.5%GN) 109 °C (PVC/2%GN)
Hasan M. et al., 2015 [107]	PVC powder (M = 1020), Yakuri Pure Chemicals, Kyoto, Japan	Mixing GN in the PVC solution-deposition to prepare the PVC/GN fibers	DSC	80 °C
	1 and 3 wt.% of GN (thickness ~8 nm and mean length ~500 nm), Iljin Nano Tech, Seoul, Korea			GN content-dependent 83.78 °C (PVC/(1%GN) 86.49 °C (PVC/(3%GN)
Salavagione H. J. and Martínez G., 2011 [108]	PVC bull polymerization at 90° C, 20% conversion	Reaction-dissolution in DMF-precipitation	DSC	81.8 °C
	5 wt.% of reduced graphene oxide (RGO), synthesized by the cited authors			84.7 °C
	1, 1.5, 2, 3, 5, and 10 wt.% of filler isocyanates-modified reduced graphene oxide (iRGO), synthesized by the cited authors			80.6–84.9 °C no dependence on concentration
	1.2 and 1.4 wt.% of RGO grafted on PVC modified by nucleophilic substitution PVC (RGO-e-PVC), synthesized by the cited authors			RGO content-dependent 102.1 °C (PVC/1.2%RGO) 106.7 °C (PVC/1.4%RGO)
Molla-Abbasi P., 2020 [109]	PVC powders with to molecular weights: 60,000 g/mol (P60) and 130,000 g/mol (P130), Sigma-Aldrich, Darmstadt, Germany	Solution casting from THF	DSC	PVC grade-dependent 79 °C (P60) 85 °C (P130)
	2 and 4 wt.% of MWCNT (diameter of 10 to 40 nm, average lengths of 1 to 25 µm, CNT 100, Korea			PVC grade-dependent 83 °C (P60/2%CNT) 87 °C (P130/2%CNT)
Mindivan F. et al., 2020 [110]	PVC, no data	Solution casting from THF	DSC	44.71 °C
	0.1, 0.3, 0.5, 1.0 wt.% of graphene nanoplatelets GNP (thickness ~5–8 nm and lateral dimension ~ 5 nm), Grafen Chemical Industries, Ankara, Turkey			GNP content-dependent 34.99 °C (PVC/0.1%GNP) 44.36 °C (PVC/1.0%GNP)
Akhina H. et al., 2020 [117]	PVC powder with K value 67 (Reliance Industries Limited, Mumbai, India) with 40% of plasticizer and stabilizers	Kneading-compression molding	DSC by use modified procedure	7 °C
	0.5, 1.0, 2.0 and 5.0 phr of RGO prepared from graphite, Merck, Darmstadt, Germany			RGO content-dependent 5 °C (PVC/0.5 phr RGO) 10 °C (PVC/5.0 phr RGO)
Li Q. et al., 2021 [111]	PVC powder with K value 64.6–66.0 (Formosa Plastics Industry, Taipei, Taiwan) with 20% of plasticizer and stabilizers	1. Mechanical activation with ball milling-compression molding (MA) 2.Conventional stirring method- compression molding (DS)	DMA tan δ	61.68 °C
	0.06, 0.13, 0.25, 0.5, 1.0, and 2.0 wt.% of graphene powder prepared from graphite, Aladdin Industrial Corporation, Shanghai, China			no dependence on concentration and processing method MA 62.01 °C (PVC/0.2%GN)–62.64 °C (PVC/1%GN) DS 62.90 °C (PVC/0.2%GN) – 62.43 °C (PVC/1.0%GN)
Pekdemir M.E., 2020 [116]	PVC powder (Mn = 63,000 g/mol), PETKIM Turkish Company, Aliaga, Turkey	Solution casting from THF	DSC	76.8 °C
	14.7% of carbon fibers, no data			86.7 °C
